# Supplementary material for: A Global Screen for Assembly State Changes of the Mitotic Proteome by SEC-SWATH-MS
Source: Cell Syst. 2020 Feb 26;10(2):133–155.e6. doi: 10.1016/j.cels.2020.01.001 (PMC7042714; doi:10.1016/j.cels.2020.01.001)

Q13263 | TIF1B_HUMAN | TRIM28 KAP1 RNF96 TIF1B
Monomer MW [kDa]: 88.55 Monomer expected elution fraction: 41

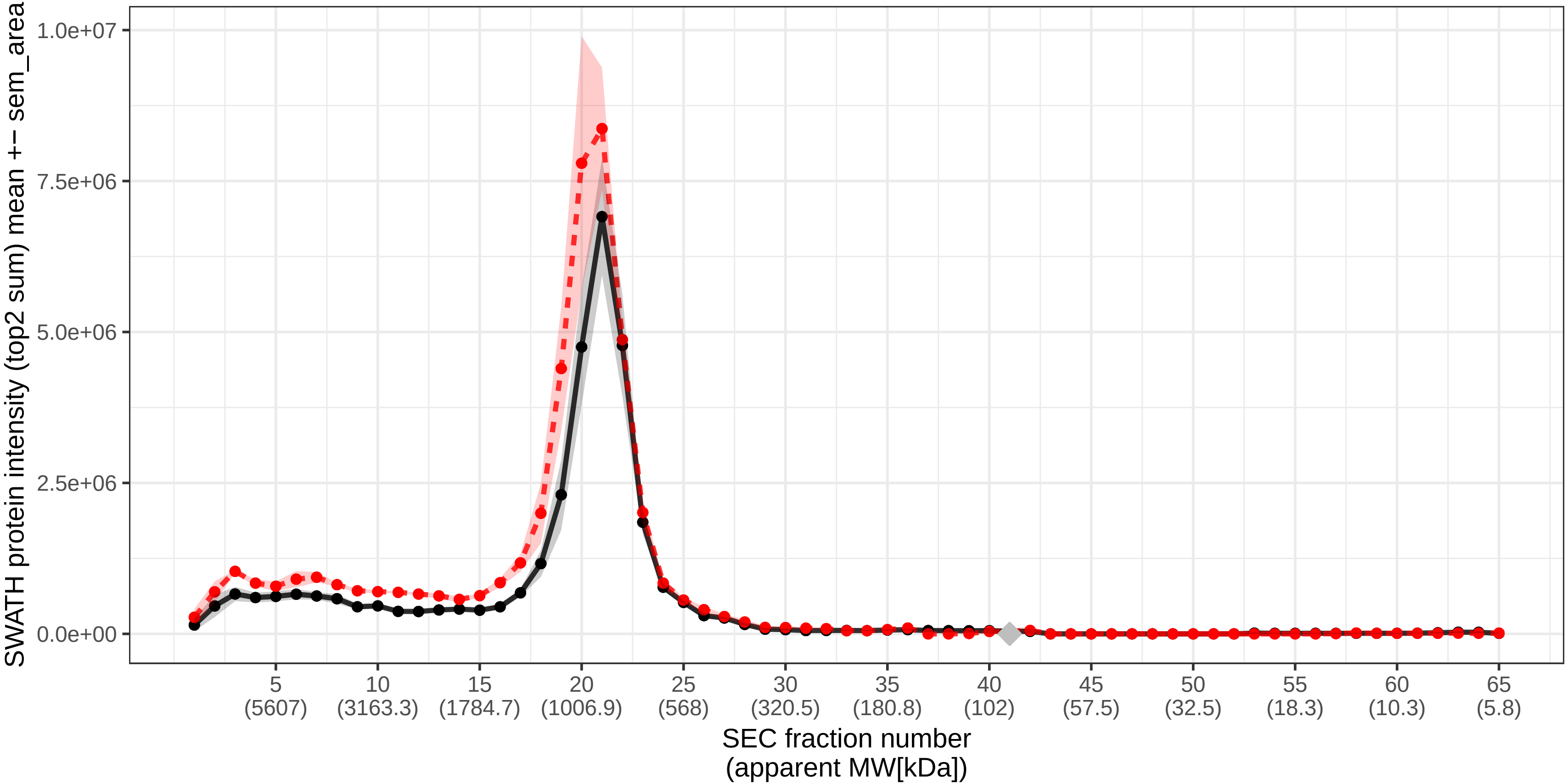

Supplement: Data S1. SEC-SWATH-MS Protein Chromatograms, Related to Figure 1 [file mmc6.zip › SECchrom_Q13263_TIF1B_HUMAN_TRIM28_KAP1_RNF96_TIF1B.pdf]
